# Recent advances and application of whole genome amplification in molecular diagnosis and medicine

**DOI:** 10.1002/mco2.116

**Published:** 2022-02-03

**Authors:** Xiaoyu Wang, Yapeng Liu, Hongna Liu, Wenjing Pan, Jie Ren, Xiangming Zheng, Yimin Tan, Zhu Chen, Yan Deng, Nongyue He, Hui Chen, Song Li

**Affiliations:** ^1^ Hunan Key Laboratory of Biomedical Nanomaterials and Devices Hunan University of Technology Zhuzhou China; ^2^ School of Early‐Childhood Education, Nanjing Xiaozhuang University Nanjing China; ^3^ State Key Laboratory of Bioelectronics Southeast University Nanjing China

**Keywords:** genomic DNA, isothermal amplification, molecular diagnosis, polymerase chain reaction, whole genome amplification

## Abstract

Whole genome amplification (WGA) is a technology for non‐selective amplification of the whole genome sequence, first appearing in 1992. Its primary purpose is to amplify and reflect the whole genome of trace tissues and single cells without sequence bias and to provide sufficient DNA template for subsequent multigene and multilocus analysis, along with comprehensive genome research. WGA provides a method to obtain a large amount of genetic information from a small amount of DNA and provides a valuable tool for preserving limited samples in molecular biology. WGA technology is especially suitable for forensic identification and genetic disease research, along with new technologies such as next‐generation sequencing (NGS). In addition, WGA is also widely used in single‐cell sequencing. Due to the small amount of DNA in a single cell, it is often unable to meet the amount of samples needed for sequencing, so WGA is generally used to achieve the amplification of trace samples. This paper reviews WGA methods based on different principles, summarizes both amplification principle and amplification quality, and discusses the application prospects and challenges of WGA technology in molecular diagnosis and medicine.

## Graphical abstract

Whole genome amplification (WGA) is a technology for non‐selective amplification of the whole genome sequence. This paper reviews WGA methods based on different principles, summarizes both amplification principle and amplification quality, and discusses the application prospects and challenges of WGA technology in molecular diagnosis and medicine.

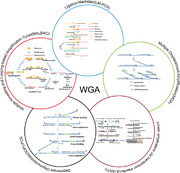

## 1 INTRODUCTION

In recent years, high‐throughput technologies such as DNA chips and NGS have greatly promoted the development and application of molecular diagnostic technology in various fields. However, the quantity and quality of nucleic acid samples are often important factors restricting the research. There are still many problems in obtaining high‐quality DNA samples as the first step of the work. WGA technology can effectively solve this problem by maximizing limited amounts of DNA as starting material while also representing the whole for high‐throughput analysis.^1^ Requiring small amounts of starting material has allowed researchers to utilize WGA technology in single‐cell research.^2^, ^3^ Single‐cell WGA principle is to amplify and sequence the whole genome DNA of the single cell with high‐throughput sequencing.^4^

WGA technology is divided into three categories: First is polymerase chain reaction (PCR)^5^ such as primer extension preamplification PCR (PEP‐PCR), degenerate oligonucleotide primer PCR (DOP‐PCR),^6^ tagged random primer PCR (T‐PCR), long and accurate PCR (LA‐PCR), ligation‐mediated PCR (LM‐PCR), interspersed repetitive sequence PCR (IRS‐PCR). The second category is isothermal amplification,^7^ such as multiple displacement amplification (MDA),^8^ multiple annealing and looping‐based amplification cycles (MALBAC),^9^ and linear amplification via transposon insertion (LIANTI).^10^ The final category is microfluidic amplification.^11^ This paper will introduce these methods from the aspects of principle and application and summarize the advantages and disadvantages of each method.^12^

## 2 PCR WGA TECHNOLOGY

### 2.1 PEP‐PCR

Among all the WGA methods, PEP‐PCR has been around the longest.^13^ PEP uses random primers and genomic DNA (gDNA) to randomly amplify the whole gDNA.^14^ The random primers are composed of 15 random nucleotides with 4 sequences.^15^, ^16^ During the amplification, the primers randomly anneal to a large number of gDNA sites starting at 37℃ and extend continuously to 55℃ to ensure that the random primers anneal to as many genomic sequences as possible.^17^ After 50 cycles, around 75% of the gDNA is amplified.^18^ Single‐cell PEP amplifies around 70% of the whole genome DNA^19^ while using random primers and less intensive PCR cycle parameters, which may lead to uneven amplification results.^20^

### 2.2 DOP‐PCR

**FIGURE 1:**
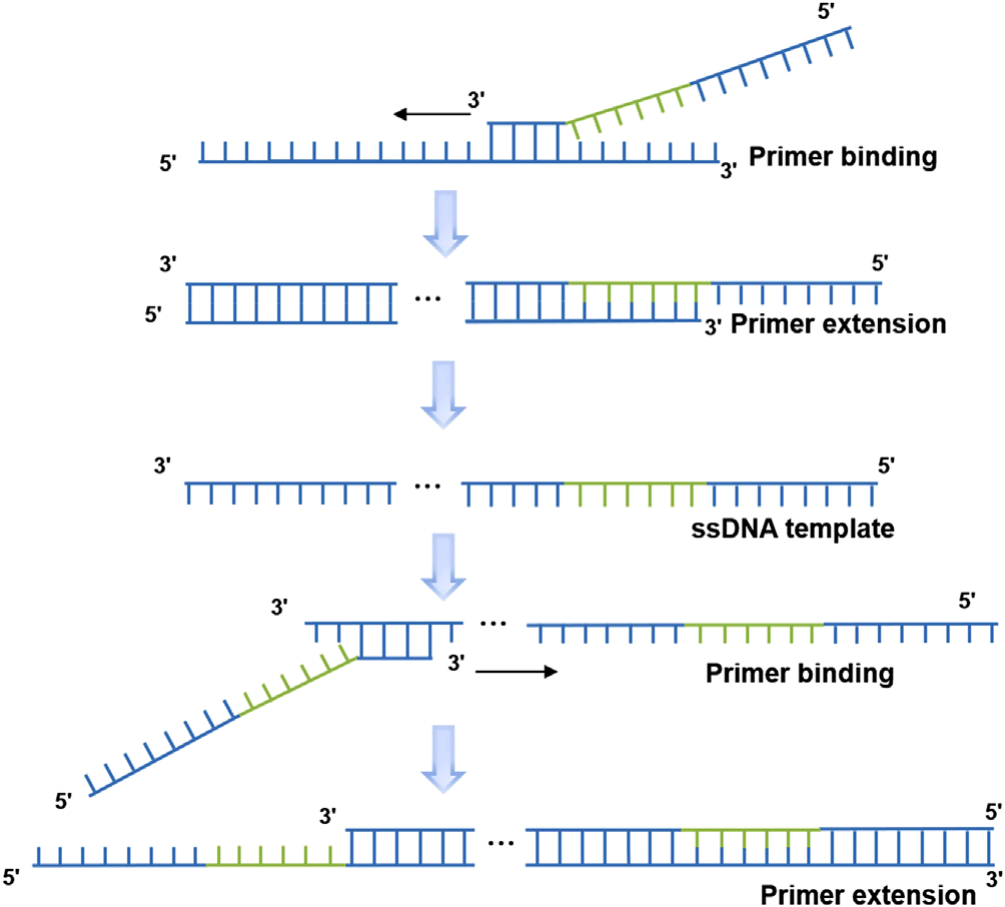
Polymerase chain reaction (PCR) reaction procedure of degenerate oligonucleotide primed PCR. The degenerate oligonucleotide primers are used in whole genome amplification. ssDNA template: single‐stranded DNA template (reproduced from Huang et al.^23^ with permission from Annual Reviews)

DOP‐PCR is commonly used for samples with limited quantity to increase the amount of DNA yielded.^21^ The schematic diagram of DOP‐PCR amplification is shown in Figure 1. DOP‐PCR uses non‐selective amplification to achieve whole genome sequencing. The principle of DOP‐PCR is similar to that of PEP, except that a fixed sequence with high frequency is added to the two ends of the six random primer sequences. The 3'‐terminal ATGTGG (A, adenine; T, thymine; G, guanine) plays a guiding role during annealing, while the six random sequences at the center play a role in strengthening the binding of primers and DNA, and the CCGACTCGAG (C, cytosine) sequence at the 5' end is used for terminal modification. DOP‐PCR consists of 50 cycles, with the annealing temperature of each cycle continuously rising, ensuring that different primers and as many genomic sequences as possible are annealed so that the whole genome can be amplified evenly in proportion.^22^, ^23^

Due to its exponential amplification, the gene coverage of DOP‐PCR technology is typically low.^24^ Any slight differences in amplification factors between different sequences will be amplified exponentially, contributing to over‐amplified and under amplified areas in the genome, ultimately giving a low coverage rate.^25^ This is due to the poor uniformity of amplification caused by exponential amplification, which worsens the coverage. However, this method can amplify an initial starting material of pg level, so when the initial amount of amplification template is very low, it can be used for pre‐amplification. Although DOP‐PCR lacks completeness in obtaining the whole genome, it can be applied to the measurement of copy number variation (CNV) in a large genome range (1 million base pairs).^26^

There are multiple factors that can influence PCR amplification. The concentration of primers, template size, guanine‐cytosine (GC) content, and DNA secondary structure will affect the amplification efficiency of polymerase and any deficiencies can affect the whole genome coverage.^27^ All of the above‐mentioned factors could lead to uneven amplification or non‐specific amplification and ultimately result in amplification bias.^28^

### 2.3 T‐PCR

T‐PCR is a WGA technology,^29^ which was first reported by Jeffreys in 1991. The 3' end of the primer is a random primer with 9–15 bases, and the 5' end is a fixed marker sequence with 17 bases. The reaction is divided into two steps: During the first step, 3' random primers are hybridized to the DNA template at 30–40°C and extended under the guidance of the Taq enzyme; in the second step, the newly synthesized DNA strand is used as a template with labeled base sequences at both ends; after 2–5 cycles, the unbound primers and primer dimers are removed by centrifugation. Finally, the PCR primers complementary to the marker sequence are added to amplify the products of the first step. Multiple replications can amplify the whole genome.^30^

T‐PCR has the advantages of high amplification efficiency and strong specificity^29^ but remains challenging to optimize the concentration of random primers.^31^ Primer dimers that are unbound are washed away in the first step.^32^ The initial template can be lost during the first washing step, and later, purification could lead to the loss of alleles. Thus, this is typically performed by a skilled technician.

### 2.4 LA‐PCR

There are two types of heat‐resistant DNA polymerase used in PCR technology^33^: The first does not contain 3'‐5' exonuclease activity, including Taq DNA polymerase, klentaq1 DNA polymerase, and pfu (exo‐) DNA polymerase; while the second contains 3'‐5' exonuclease activity, including pfu DNA polymerase, Vent DNA polymerase, and Deep Veep DNA polymerase, among others. The thermostable Taq DNA polymerase commonly used in PCR has a strong elongation ability, but it cannot correct the mismatched bases introduced in the process of amplification. The mismatched bases at the 3' end affect the smooth progress of the extension in the amplification process^34^ so that the extension terminates ahead of time, thus limiting the length of the amplification fragment. Pfu DNA polymerase can replace Taq DNA polymerase (the fidelity of pfu DNA polymerase is 5–10 times higher than that of Taq DNA polymerase) while significantly reducing the production of mismatch bases, although studies have shown that a polymerase with exonuclease activity,^35^ such as pfu, Vent and Deep Vent, cannot effectively amplify long fragments alone. This may be due to the enzyme degrading the primers during the long‐term amplification process due to the 3'‐5' exonuclease, which has high activity. Only the low level of 3'‐5' exonuclease activity can successfully amplify the long fragment. Based on this principle, a high level of thermostable DNA polymerase without exonuclease activity and a small amount of DNA polymerase with exonuclease activity were mixed, and the extension ability of the former and the correction ability of the latter were used to successfully amplify the long fragments of the two kinds of enzymes under appropriate proportions and reaction conditions. Presently, the mixed enzymes used in LA‐PCR include thermostable deoxyuridine 5'‐triphosphate pyrophosphatase (dUTPase) in addition to the above two enzymes, one enzyme does not contain 3'‐5' exonuclease activity and the other enzyme contains 3'‐5' exonuclease activity.^36^ The deamination of deoxycytidine triphosphate (dCTP) will occur in the process of PCR and deoxyurodine triphosphate (dUTP). DNA polymerase with correction function irreversibly binds to deoxyuracil in vitro, which affects the efficiency of the enzyme and leads to the failure of LA‐PCR amplification. dUTPase will degrade dUTP, and effectively prevent dUTP from participating in the synthetic DNA during the PCR process. Hogrefe et al. found that heat‐resistant dUTPase can significantly improve the efficiency of pfu DNA polymerase amplification.^37^ Hot start enzyme^38^ has been widely used in LA‐PCR.^39^ Hot start helps prevent the non‐specific amplification caused by the mismatch of primers or the formation of primer dimer in the PCR reaction, thus improving the amplification efficiency of target DNA fragments.

LA‐PCR technology overcomes the shortcomings of traditional PCR technology, such as short amplification fragment (often below 3 kb) and low fidelity. Its amplification length can exceed 10 times of traditional PCR technology and can survive longer reaction times.^40^

### 2.5 LM‐PCR

**FIGURE 2:**
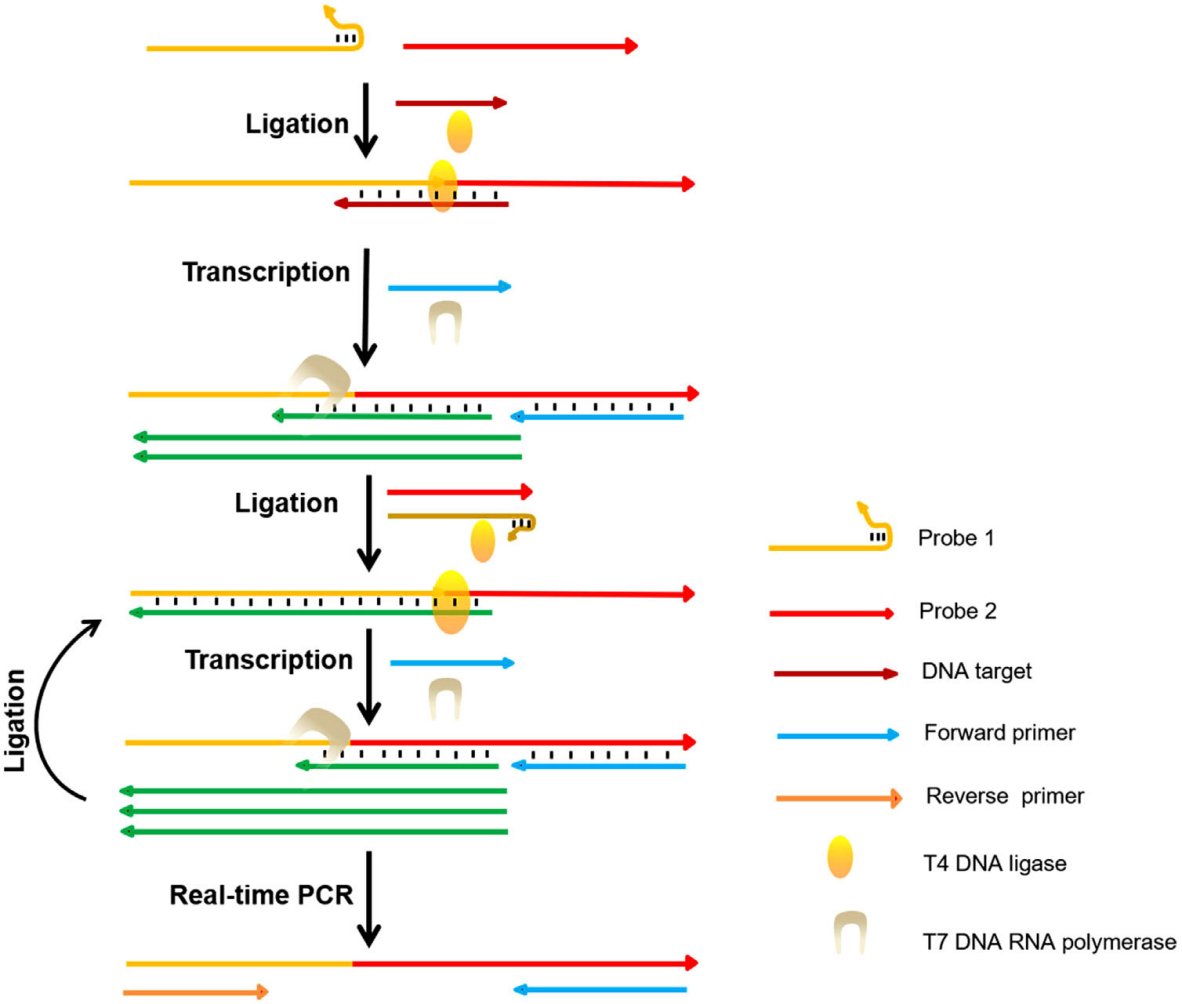
Short‐length DNA detection by using T7 RNA polymerase with LM‐PCR (reproduced from Yu et al.^44^ with permission from American Chemical Society)

LM‐PCR is a unilateral PCR technique^41^ consisting of five steps. The first step uses chemical cleavage to cleave gDNA at specific sites^42^ to produce 5'‐phosphate molecules. The second step uses the DNA fragments produced in the first step as a template and uses primer extension reactions guided by genome‐specific primers to produce flat‐end DNA molecules. During the third step, a common linker connects to the flat‐end DNA molecule produced above. The fourth step uses genome‐specific second primers and linker primers for PCR amplification of the ligated products. Finally, the labeled genome‐specific third primer was used to extend the PCR product, followed by visual electrophoresis and sequencing. The primers or probes can also be engineered to bind T7 RNA polymerase transcription to ligation as shown in Figure 2. Thus, a combined DNA strand consisting of two DNA probes^43^ can be used as a transcription template reaction following the PCR process shown in Figure 2.

LM‐PCR generally adopts the method of three primers, and the connection between the three is that the extension of the second primer is located at the 3' end of the first primer, and so is the connection between the third primer and the second primer. This design greatly enhances the specificity of the reaction.^45^

When using LM‐PCR for in vivo foot printing or sequencing, we are most concerned about the following three aspects: sensitivity, fidelity, and convenience of primer design. According to the principle of LM‐PCR, only when the third labeled primer competes effectively with the second primer in the PCR reaction, and annealing of PCR products template, the PCR product can be efficiently labeled by the labeled primer. When designing the third marker primers, a partial overlap should be left between the third primer and the second primer, and the melting temperature (Tm) value of the third primer should be higher than that of the second primer. This requirement greatly limits the design of PCR primers in the application of this technique,^46^ especially when studying the interaction between DNA proteins rich in adenine‐thymine (AT) regulatory sequences and determining the nucleotide sequence.

### 2.6 IRS‐PCR

IRS is a widely distributed and highly conserved interval repeat sequence in gDNA.^47^ The primer synthesized IRS‐PCR amplifies DNA between two IRS sequences. Alu sequence is a human unique IRS sequence, which contains about 900,000 copies in the human haploid genome, with an average interval of 4 kb. Due to the uneven distribution of Alu sequences in the human genome and different repetition times, the amplification efficiency of Alu‐PCR varies greatly in different regions of the genome, causing uneven amplification products, which limits its application.

## 3 ISOTHERMAL AMPLIFICATION TECHNOLOGY

### 3.1 MDA

**FIGURE 3:**
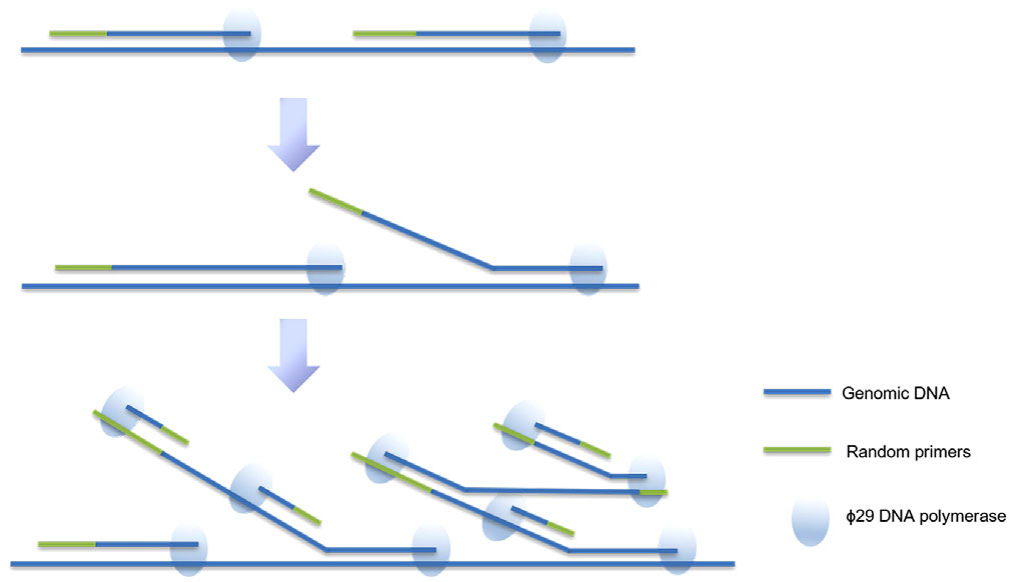
Multiple displacement amplification of genomic DNA. This reaction is an isothermal amplification reaction using random primers for φ29 DNA polymerase. (reproduced from Huang et al.^23^ with permission from Annual Reviews)

MDA is a WGA technique developed in recent years. It is the most popular method of whole genome isothermal amplification at present.^48^ MDA uses DNA polymerase with high extension activity and random primers resistant to exonuclease to amplify the genome under isothermal conditions. This method is based on chain substitution and was originally used to amplify huge circular DNA such as a plasmids or bacteriophage DNA. Now it is also used to amplify linear DNA.^49^ As shown in Figure 3, it can uniformly amplify the whole genome with high fidelity, amplify 10 to 100 kb sized fragments, and provide a large number of uniform and complete whole genome sequences.^50^ It can be amplified by random primers resistant to exonuclease activity and template annealing under the action of φ29 DNA polymerase with strong strand replacement activity and exonuclease activity.^51^

Φ29DNA polymerase has a strong error correction function, and the amplification product has high fidelity, which ensures the consistent yield of different templates.^52^ The longest amplification product is 100 kb, with an average of more than 10 kb. Using a 100 μl reaction system, the amplification product can be kept around 20–30 μg, which can be used for many aspects of DNA analysis.^53^ MDA is simple to operate, reduces human error, avoids DNA loss, and is available in commercial kits. Although some studies have found that when used with low concentration DNA starting template, it has some shortcomings, such as poor repeatability, allele loss, and inconsistent location.^54^

To verify the advantage of MDA in WGA, researchers have demonstrated the overall effectiveness of MDA in maintaining the quality of sequencing data and the abundance of species measurements in eight paired metagenomic samples and one titrated mixed control sample,^55^ while providing higher high‐fidelity DNA yields and increasing the number of high fidelity DNA in low biomass samples.^56^

Uneven amplification in MDA causes a lot of problems with biological applications of copy number estimation.^54^ Under optimized conditions, related studies have proved that the whole reaction system can be physically separated into many microchambers or droplets using microfluidic devices, which can effectively reduce this deviation.^57^ This improved MDA technique, also known as micro‐channel MDA (μCMDA), allows MDA reagents to be dispersed into one‐dimensional tubules. Due to the dual effects of soft segmentation of high molecular weight DNA molecules and limited diffusion of small particles,^58^ μCMDA shows significant effectiveness in improving amplification uniformity. μCMDA can effectively improve our efficiency and sensitivity in detecting single nucleotide variants (SNVs).^59^ Additionally, this simple method requires neither customized instruments nor complex operations and has gradually developed into an off‐the‐shelf technology in almost all biological laboratories.^60^

It is also possible to sequence the DNA of a single cell by using expanded DNA as a template. MDA amplification can amplify a few micrograms of DNA in a bacterium to micrograms of DNA^61^ that can be used for sequencing. It is currently recognized as the best single‐cell genome amplification technology.^60^ As a great breakthrough in environmental research methods, MDA can be used to guide new research strategies in microbial genetics, ecology, and infectious diseases research.^62^

Previously, newly discovered microbes can only be sequenced by growing enough strains to provide enough DNA templates. However, less than 1% of microbes have been successfully cultured. A large number of new unculturable organisms will appear in the natural environment, which will become a major obstacle to the traditional genome research methods. In this work, the emergence of single‐cell sequencing has opened up a new field in which DNA templates can be obtained directly from a single cell without the need for improved culture methods.^63^ Using MDA, amplified DNA from single‐celled microorganisms can be sequenced. The high molecular weight DNA of micrograms can be amplified from several Feike (10–15 g) DNA in bacteria. The DNA amplified by high molecular weight DNA is suitable for preparing DNA libraries and Sanger sequencing. The DNA generated by MDA can also be directly used as a template for pyrophosphate sequencing. The linkage of gene sequences between individual cells is a powerful tool, which can be used to guide the construction of gene chips for multiple organisms obtained from extracted environmental DNA (macro genomic sequence) by shotgun sequencing.^49^

The MDA method has a higher genome coverage than DOP‐PCR.^62^ The high efficiency and high fidelity of φ29DNA polymerase allows MDA to possess significant advantages in the analysis of SNV and the construction of large fragment libraries. However, like DOP‐PCR, MDA is still an exponential amplification; thus, sequence preference of PCR response is still present.^64^ However, unlike DOP‐PCR, this preference cannot be repeated, thus causing the MDA method to be unsuitable for CNV analysis.

### 3.2 MALBAC

**FIGURE 4:**
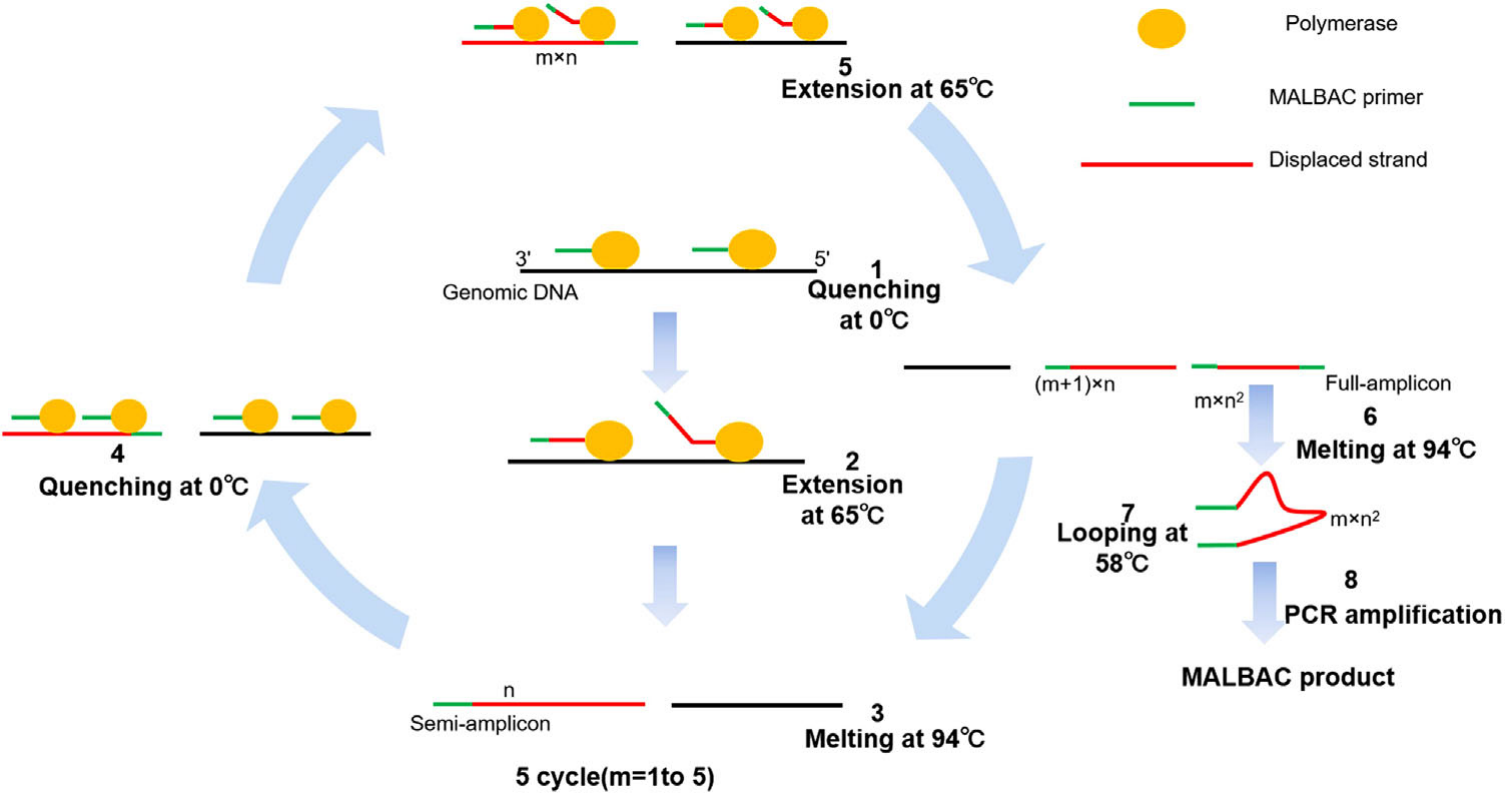
The schematic diagram of multiple annealing and looping‐based amplification cycles. *m*: the number of temperature cycles; *n*: the number of primers binding; (*m* + 1) × *n*: the number of semi‐amplicons appear at the *m*th cycle; *m* × *n* ^2^: the number of full amplicons produced in the *m*th cycle (reproduced from Huang et al.^23^ with permission from Annual Reviews)

MALBAC, shown in Figure 4, is the most advanced WGA technology at present. This technology can complete the high‐precision whole genome sequencing of a single cell.^65^ During MALBAC, five rounds of MDA pre‐amplification are carried out to make the amplification products form closed ring molecules. Because the ring molecules cannot be further amplified, the amplification process becomes a linear amplification, and conventional PCR amplification is carried out. The template used at this time is more uniform, thus obtaining amplification products with less amplification offset and sufficient coverage.^23^ Amplifiers complement each other and form a loop, preventing an exponential amplification of DNA, and reducing amplification bias so that 93% of the genomes in a single cell can be sequenced. This technique has high sensitivity, thus allowing a single cell and single chromosome or 0.5 pg gDNA to be amplified. The fragment length is mainly distributed among 0.8–10 kb, and the amplification uniformity is better than other WGA techniques, which is satisfied with a variety of downstream applications.^50^

MALBAC makes it easier to detect smaller DNA sequence variations in a single cell, so genetic differences between individual cells can be found. Such differences can help explain the mechanism of cancer progression, the formation of germ cells, and even the differences of individual neurons. Although it still has a certain sequence preference, unlike MDA, MALBAC is repeatable between different cells, so CNV analysis can be carried out after standardizing the reference cells.^66^ In addition to its repeatability, the knockout allele rate of MALBAC amplification is lower due to the homogeneity of its amplification. However, the fidelity of polymerase used in this method does not perform as well as φ29,^67^ allowing the potential of more false positives in the detection of SNV. Also, low amplification regions on the genome are sometimes lost in the process of amplification due to its repeatable sequence preference. At present, MALBAC technology has been successfully applied to human single sperm,^68^ blastocysts and polar bodies screened before implantation,^9^ early developmental embryos,^69^ tumor cells,^70^ trace material evidence, and microorganisms at criminal investigation sites.^50^

Under the same conditions, the genome coverage of MALBAC is higher and more uniform than MDA,^71^ and at the same time, producing fewer false positives and maintaining higher accuracy. In addition, there is some difference between the two methods in variation detection. The identification of single nucleotide polymorphisms (SNPs) in sequenced samples revealed that more high‐quality SNPs were detected in MALBAC samples than in MDA samples, and the analysis showed that the MALBAC method had better performance in terms of uniformity and reproducibility of the whole genome amplification.^71^

### 3.3 LIANTI

**FIGURE 5:**
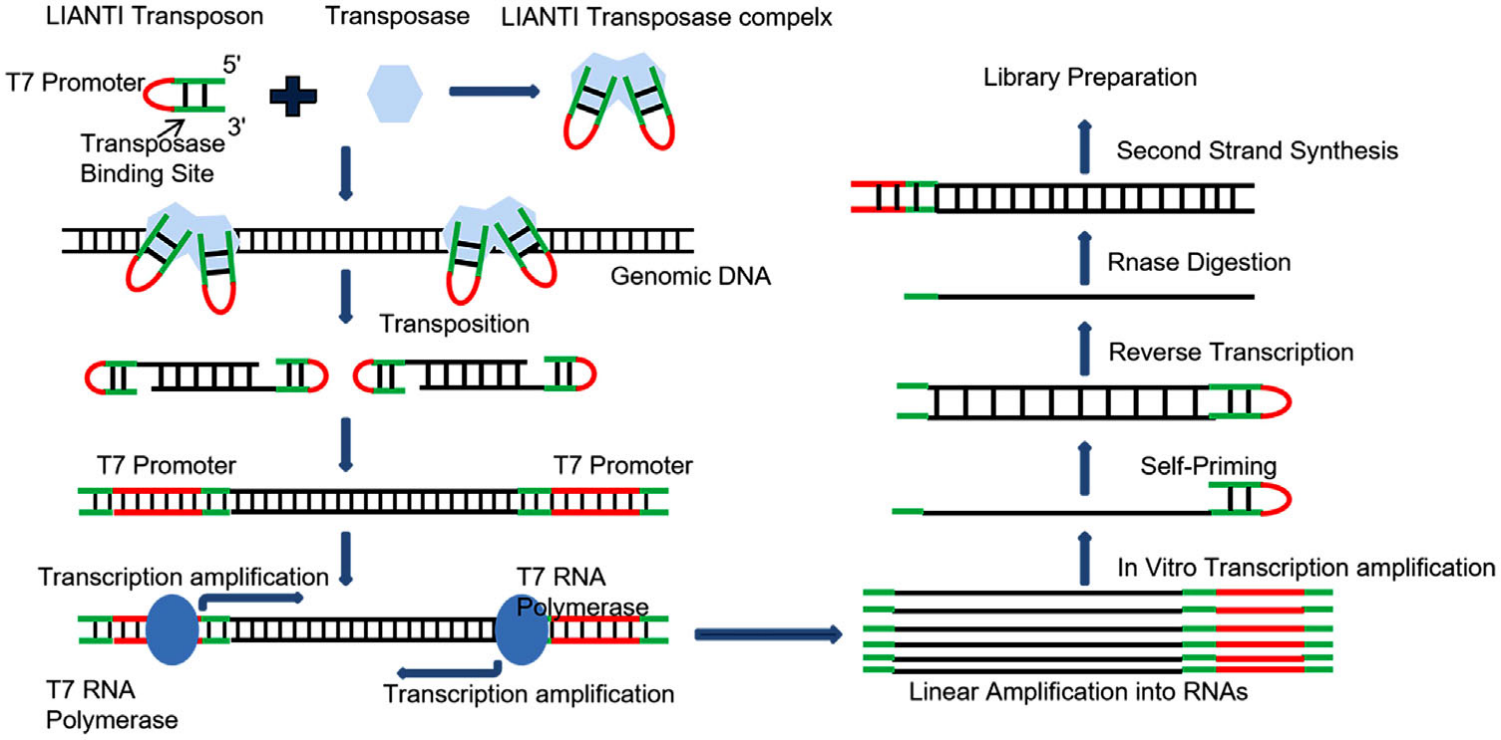
The schematic diagram shows the amplification principles and sequencing library preparation of the linear amplification via transposon insertion method (reproduced from Li et al.^74^ with permission from MDPI)

LIANTI is a new WGA method for single‐cell sequencing reported by Xie in *Science 2017*.^10^ LIANTI can obtain adequate DNA through linear amplification, reducing errors caused by exponential amplification and non‐specific in MDA, MALBAC, and other methods.^72^ The main differences between primary WGA methods are shown in Table 1. This amplification method is an improved single‐cell WGA technique based on the traditional methods, linearly amplified by the transposon. The transposon is a DNA that can change its position in the genome. It has a double‐stranded transposase‐binding site of 19 base pairs and a single‐stranded T7 promoter loop. The promoter is part of the DNA that starts the transcriptional process and is used to amplify downstream DNA and prepare DNA libraries.^73^ First, Tn5 transposon was combined with LIANTI sequence to form Tn5 transposable complex with T7 promoter and then randomly inserted into gDNA.^74^ After transposon, DNA was randomly fragmented. Subsequently, the T7 promoter performed in vitro transcription function to obtain a mass of linear amplified transcripts. After reverse transcription, substantial amplified products are obtained and DNA is ready for library preparation and sequencing as shown in Figure 5. There is no exponential amplification and non‐specific primers in the LIANTI process, which avoids the interference of PCR, dramatically reduces the amplification deviation and error, and improves the amplification stability.^75^ The LIANTI is superior to the existing WGA methods. It can detect micro CNVs at kilobase resolution, which can be used to observe the random initiation of DNA replication from cell to cell. In addition, LIANTI is more accurate than MDA and MALBAC in detecting CNVs and SNVs.^76^

**TABLE 1:** Differences between whole genome amplification methods

	**PEP‐PCR**	**DOP‐PCR**	**LM‐PCR**	**T‐PCR**	**MDA**	**MALBAC**	**LIANTI**
Principle	Completely random priming method	Partial random priming method	Adaptor ligation‐mediated PCR	Two‐step PCR method using tagged random primer	Multiple displacement amplification	Multiple annealing and looping‐based amplification cycles	Using Tn5 transponson to achieve amplification without the non‐specific primers
Primer	Random primers containing 15 bases	Degenerate primers containing six random primers	Universal primer and an adaptor primer	Tagged random primers containing a 9 to 15 bp arbitrary 3' tail that can bind to any DNA sequence	Six random primers	Twenty‐seven universal primers and eight random primers	/
Enzyme	DNA polymerase	DNA polymerase	Taq DNA polymerase	Taq DNA polymerase	Phi29 DNA polymerase	Bst enzyme; Phi29 DNA polymerase	T7 RNA polymerase
Coverage	∼40%	∼50%	∼96%	∼37%	∼70%	∼90%	∼97%
Uniformity	Low	Medium	Low	Medium	Low	High	High
Coefficient of variation	Medium	High	High	High	Medium	Medium	Low
GC preference	High	High	High	High	Medium	Low	Low
Advantages	The operation is simple, the quality of template DNA is low, the minimum starting template quantity is up to 5 pg	Simple to operate, minimum starting template up to 50 pg	The yield is high, the fragment is long, and the quality of template DNA is low	High amplification efficiency and product specificity	High yield, minimum initial amount up to 10 pg, good fidelity	Simple operation, high output, minimum starting template of several pocks, reliable and repeatable results	Small amplification deviation and high gene coverage
Disadvantages	Low output and poor fidelity	The amplification deviation is large when the initial template is very low	The operation is tedious, and the template DNA is easy to be lost by multi‐step operation	Low gene coverage	Large amplification deviation	It is more difficult to amplify when the initial template is very low, and it is easy to appear false positive	Prone to false positives
Application	LOH analysis, STR analysis, and so forth	FISH, SNP analysis, SSCP analysis, and so forth	CGH, SNP, STR analysis, Library establishment, Gene detection, and so forth	STR analysis, Forensic Medicine, DNA identification, and so forth	SNV detection, NGS, STR analysis, single‐cell sequencing, and so forth	Chromosome analysis, CNV detection, SNV detection, CGH, single‐cell sequencing, and so forth	CNV detection, SNV detection, single‐cell sequencing, haploid typing, and so forth
Reference	^86^	^9^, ^87^, ^88^	^44^, ^89^, ^90^, ^91^	^44^	^17^, ^28^	^4^, ^83^, ^92^	^93^, ^94^

Abbreviations: CGH, comparative genomic hybridization; DOP‐PCR, degenerate oligonucleotide primer PCR; FISH, fluorescence in situ hybridization; LIANTI, linear amplification via transposon insertion; LM‐PCR, ligation‐mediated PCR; LOH, loss of heterozygosity; MALBAC, multiple annealing and looping‐based amplification cycles; MDA, multiple displacement amplification; PCR, polymerase chain reaction; PEP‐PCR, primer extension preamplification PCR; SSCP, single‐strand conformation polymorphism; STR, short tandem repeat; T‐PCR, tagged random primer PCR; SNP, single nucleotide polymorphism; SNV, single nucleotide variant; CNV, copy number variation; NGS, next‐generation sequencing.

However, LIANTI still has some limitations. It is highly dependent on Tn5 transposase and has not yet achieved large‐scale application. Moreover, its experimental operation is more tedious and lacks repeatability.^77^ Therefore, the future development of LIANTI needs to be further explored.

## 4 MICROFLUIDICS‐BASED AMPLIFICATION TECHNOLOGY

In recent years, the progress of methodology has extensively promoted the development of molecular diagnosis technology. However, there are often many disadvantages in using traditional macro detection platforms for microscopic molecular level characterization and analysis, such as complex instruments, serious interference, complicated operation, easy cross‐contamination and poor stability. The timely emergence of microfluidic opens a new door for the research of molecular diagnosis.^78^

Microfluidic technology refers to the science and technology of precise manipulation, analysis, and detection of a microfluidic or micro reaction system in the micron flow channel. Its core unit is the microfluidic chip.^79^ The microfluidic chips can integrate complex fluid control systems and functional unit modules on a few square centimeters of a chip, with the benefits of automation, integration, miniaturization, and so on.^80^ Therefore, it is called a micro‐complete analysis system or chip laboratory. The combination of microfluidic technology and micro reaction system has the advantages of small sample size, fast reaction speed, high sensitivity, and flexible manipulation. At the same time, the closed microfluidic chip can avoid cross‐contamination of the sample and reduce biohazard, which has a natural advantage in molecular diagnosis.^81^, ^82^ The method based on microfluidic technology provides an ideal platform for nucleic acid amplification. The nucleic acid amplification on the chip mainly depends on the rapid prototyping of polydimethylsiloxane or glass etching, which has the advantages of fast amplification speed, low detection limit, and high detection accuracy.

The amplification methods mentioned above, such as DOP‐PCR, MDA, and MALBAC, are all at the molecular level. The process of combining high fidelity DNA replication or linear amplification steps helps improve consistency, expand coverage, and reduce the error rate. Among these methods, MDA is easy to operate and provides higher fidelity and coverage. However, they all need to amplify the genome in vitro with DNA polymerase and then prepare a library for high‐throughput sequencing with a relatively short read length. The short sequence reads can affect the acquisition of gene information, especially for single‐cell research. Therefore, Xie et al.^4^ reported the emulsion WGA technique in 2015, which combines emulsion, MDA, and microfluidic chip to accurately detect the genomic variation. This method showed significant improvement in the uniformity of amplification, the coverage of the genome, and the proportion of false positives. Later, Zhang made a further improvement based on this method. Namely, large fragments are evenly distributed into 24 independent reaction cells to amplify and prepare the library.^83^ Single cells were lysed in a tube and then mixed with MDA reaction buffer. The emulsification used in the microfluidic cross‐connection device generates uniformly distributed droplets for DNA amplification, which maintains the high fidelity of MDA and improves the uniformity of amplification, which has attracted extensive attention in various single‐cell studies.^84^, ^85^

However, these microfluidic‐based methods still face various difficulties to meet the requirements of WGA. For example, these methods are both difficult to operate and to control droplets in parallel, limiting capturing the required droplets. In addition, a large number of monodisperse droplets are usually unstable, which may affect the uniform amplification of each droplet DNA fragment. Another disadvantage is that the droplet‐based method cannot be automated for WGA in integrated microfluidic chips. However, the market demand for molecular diagnosis and the continuous progress of microfluidic technology will promote the development of microfluidics in molecular diagnosis. With these advancements in microfluidics, advanced molecular diagnostic products based on microfluidics will appear centrally to provide better services for human health.

Based on PCR amplification, DOP‐PCR uses universal primers on both sides of the site to amplify the sample.^95^ The detection accuracy of single‐cell CNV is high, but the coverage of calling SNV is low, creating higher false positive and negative rates.^96^ The coverage of MDA has greatly improved, but the sensitivity in CNV determination is often low because its amplification gain varies along the genome and cannot be reproduced between different cells.^97^ Through quasi‐linear amplification, MALBAC suppressed the random deviation of amplification, reduced the rate of allelic dropout, and produced a lower false negative of SNV detection.^68^ Despite its shortcomings, MDA still provides genomic coverage equal to or higher than that of MALBAC in both individual diploid cells and aneuploid cells, such as mitotic^98^ and cancer cells.^61^ The main advantage of MDA is its low false‐positive rate of SNV detection, which is due to the use of pHi‐29, a highly progressive high‐fidelity polymerase. Microfluidic devices have been implemented for single‐cell WGA,^99^ allowing contamination avoidance and parallel high‐throughput analysis of multiple single cells. The total reaction volume of the microfluidic device is very small (up to nanoliters or picoliters), which not only promotes the reaction efficiency but also greatly reduces the cost of the enzymes and reagents used. It is reported that in the WGA of a single bacterial cell, compared with the microliter device, the nanoliter volume of the microfluidic device improves the uniformity of amplification.^100^

## 5 APPLICATION OF WGA TECHNOLOGY

WGA technology has a broad application prospect for applications with limited starting material such as forensics, paleontology, prenatal diagnosis for which only a single cell can be obtained from maternal peripheral blood and amniotic fluid, and tumor molecular pathology for genetic analysis of multiple loci.

### 5.1 Application of WGA in prenatal diagnosis

Prenatal diagnosis is used to advance the diagnosis of genetic diseases and congenital malformations before the fetus’ birth and to provide sufficient and reliable information for families at high risk of genetic diseases and other factors causing malformations.^101^ To avoid the spread of pathogenic gene variation and detect genetic diseases as soon as possible, prenatal gene testing is provided.^102^ Similarily to abnormal fetal development testing by ultrasound, genetic testing may provide an accurate diagnosis so that family members can make appropriate choices for abnormal fetuses during pregnancy and take positive and effective measures to correct and reduce the birth of defective babies.^103^ However, in prenatal diagnosis, the concentration of DNA extracted is often too low due to oligohydramnios, few cells in amniotic fluid, or too many fetal fat components, which cannot be used for genetic detection.^104^ At this time, it is necessary to improve the quality of DNA through WGA technology to meet the detection requirements and amplify the whole genome of the qualified DNA quality control. For example, myotonic dystrophy type 1 can be diagnosed prenatally by WGA, using triplet‐primed PCR to detect amplified *DMPK* alleles. Using single‐tube haplotype analysis, 12 closely linked microsatellite markers with high polymorphism can be analyzed, thus avoiding the transmission of the disease to the next generation.^105^ WGA can also amplify the whole genome from a single cell, provide a new path for prenatal diagnosis, obtain a large number of DNA sequences without changing genetic information, and provide a more accurate diagnosis for clinical work.^106^

### 5.2 Application of WGA in pre‐implantation genetic diagnosis (PGD)

PGD refers to the genetic analysis of embryos before implantation.^107^ Individual blastomere cells, polar bodies, or several trophoblast cells are taken from embryos cultured by in vitro fertilization for genetic examination.^108^ PGD analysis can prevent the occurrence of genetically abnormal pregnancy and advance prenatal diagnosis before the embryo is implanted into the endometrium, to avoid repeating abortion or induced labor damage to pregnant women.^109^

At present, PCR is widely used in PGD and has sufficient detection sensitivity, but it is only suitable for specific sequence amplification and cannot meet the needs of multi‐site complex amplification.^110^ The material source of single‐cell DNA detection is limited,^111^ and the time requirement of the test is very high; if there are not enough samples, it is difficult to meet the needs of rapid detection, but WGA technology can meet the needs of this aspect.^112^ Since the first appearance of PEP and DOP‐PCR in the early 1990s, WGA has made remarkable progress in the accuracy, efficiency, and reliability of single‐cell DNA amplification and has gradually developed into a powerful tool for single‐cell gene diagnosis.

Akash et al.^113^ have verified a method to infer the whole genome sequence of embryonic inheritance for PGD. They combined haplotype analysis of parental genome sequencing with rapid embryo genotyping to predict the whole genome sequence of a fifth‐day human embryo from a couple at risk of alpha‐thalassemia. They found that genetic prediction had about 3 million paternal or maternal heterozygote sites with an accuracy of more than 99% and successfully staged and predicted the spread α1‐globin gene/α2‐globin gene (HBA1/HBA2) deletions from each parent. The experimental results show that pre‐implantation whole‐genome prediction is helpful to the comprehensive diagnosis of embryonic diseases with a known genetic basis.

In related studies, PCR and MDA+PCR were compared in the detection of PGD α‐thalassemia. The results showed that the total diagnosis rate of the MDA+PCR group (96.51%) was higher than that of the PCR group (86.16%). The rate of embryos available for transplantation in the MDA+PCR group (71.62%) was also significantly higher than that in the PCR group (62.23%), compared with the PCR group, the misdiagnosis rate was significantly lower.^114^ The whole genome of single‐cell blastomere was amplified by MDA and analyzed by fluorescent PCR, which could effectively improve the accuracy of PGD in the diagnosis of Duchenne muscular dystrophy (DMD). This is also the first successful clinical application of MDA technology in the treatment of DMD in PGD.^115^ An MDA‐PGD scheme for Marfan syndrome was designed by using MDA technology, which can be used for PCR analysis of five different sites of MDA products, and any known gene defects can be diagnosed with standard methods and conditions.^116^ Lee et al. also evaluated the value of MDA in the diagnosis of fragile X syndrome PGD.^117^ The fragile X mental retardation‐1 CGG repeat sequence, amelogenin, and two polymorphic markers were detected by fluorescent PCR, and 10 normal embryos, four mutant embryos, and six heterozygous carriers were diagnosed successfully, indicating that MDA and fluorescent PCR can be successfully used in the PGD of fragile X syndrome. This micro‐DNA amplification method can improve the sensitivity and reliability of PGD to complex monogenic diseases. PGD can also be used for monogenic disorders. The diagnostic efficiency can be improved by analyzing free gDNA in embryo culture fluid collected at the blastocyst stage. This requires a highly specific and efficient amplification method to provide sufficient samples for accurate diagnosis. WGA is considered a promising approach.^118^

### 5.3 Application of WGA in tumor

Gene amplification in tumor cells is the main mechanism of oncogene activation.^119^ Oncogene activation can cause tumor cells to escape growth inhibition and produce drug resistance.^120^ As a genetic disease, gene diagnosis and analysis of cancer individuals are essential for the guidance and realization of individualized therapy but proves difficult to achieve the therapeutic purpose by amplifying fragments from sample sites, which may be due to the small number of DNA isolated.^121^

Daisuke et al. used the MDA technique to amplify the whole genome DNA from plasma samples. They amplified two microsatellite markers, D1S243 and D19S246, thus showing microsatellite changes in patients with head and neck cancer.^122^ DNA can also be extracted from a single lesion of prostatic intraepithelial neoplasia (HPIN) and prostate cancer (CaP) and can then be amplified by MDA for gDNA array comparative genomic hybridization (GaCGH). The combined detection of laser capture microdissection, MDA, and GaCGH is very suitable for identifying abnormal chromosome copy number abnormalities (CNAs) from small cell clusters. This detection can identify potential genomic markers and locate tumor suppressor genes or oncogenes that have not been previously reported in HPIN and CaP.^123^ A microfluidic chip technology based on WGA technology has also been proposed, which can be used for whole genome sequencing of single‐cell circulating tumor cells (CTCs).^124^ It can realize the whole process from blood filtration, enrichment, identification, and cleavage to WGA, providing a solid foundation for the whole genome sequencing of CTC and the precise treatment of cancer.^125^ The progress of WGA technology in regards to gene amplification in tumors^126^ will continue to play an important role in the diagnosis and treatment of cancers.^127^, ^128^

### 5.4 Application of WGA in forensic genetics laboratories

In recent years, the research on the amplification of trace DNA samples by WGA technology has become more and more indepth.^129^ This technique can be used to amplify the whole genome of trace residual tissues, even single cells, and provide sufficient DNA templates for follow‐up multigene, multilocus analysis, and comprehensive genome research.^130^ This allows testing of precious samples with small amounts of DNA or a large number of degraded DNA in medical biology.^131^ WGA technology is particularly important to obtain a sufficient amount of DNA from a limited number of forensic samples for a large number of genetic analyses and tests.

B‐lymphoblastoid cells from female and nucleated cell samples from male venous blood have been genotyped at forensic sites with capillary electrophoresis and massively parallel sequencing (MPS)^132^ platforms to assess their performance without WGA. The study found that in the absence of WGA, except for three and five samples of female cell samples with an accuracy of more than 25%, the rest had an accuracy of well under 10%. In male samples, the Y allele was promoted, occasionally leading to relatively high mean relative fluorescence units, while WGA combined with the MDA strategy performed relatively well on STR and SNP genotyping of low copy number samples on both CE and MPS, even for single‐cell samples.^133^

The most common way to obtain DNA from forensic scenes is to extract DNA from evidence samples. A good DNA extraction method is the primary guarantee to obtain accurate genotyping results.^134^ Different extraction methods will affect the accuracy of typing results. Ballantyne et al.^135^ used the Chelex method to extract DNA. The amplification amount of Genome Plex WGA kit is more than that of the chemical extraction method. Erin et al. developed an improved PEP‐PCR. As a new WGA technique, it can effectively amplify DNA samples with low copy number (gDNA<100 pg) or environment‐induced degradation in evidence items, and can be used for subsequent microsatellite analysis.^136^

Presently, WGA technology is only a technical method to improve the initial template DNA. To overcome the difficulty of forensic trace sample testing, it is necessary to combine WGA technology with other forensic DNA testing techniques to overcome the obstacles in forensic research.^137^

## 6 CONCLUSION

In conclusion, WGA is a technology that can greatly maximize the original template DNA and provides a way to obtain a large amount of genetic information from trace gDNA.^138^ In this paper, we reviewed multiple WGA methods along with their amplification principles, advantages, and disadvantages. WGA's disadvantages consist of amplification bias,^28^ non‐specific amplification,^27^ low sensitivity, poor repeatability, a high error rate of operation, and external contamination.^139^ Researchers are continuously improving accuracy, coverage, convenience of amplification, and high sensitivity in trace samples.^140^

At present, WGA has shown strong advantages in the microanalysis of sperm, fetal cells, lymphocytes, tissue sections, and other specimens. However, reducing or even avoiding the illusion of amplification in a small number of specimens will be an important topic in the future.^141^ In addition, the application of DNA amplification in the body fluid of tumor patients, allows for a noninvasive diagnosis of tumor and amplification of processed DNA such as DNA methylation, reverse transcriptional cDNA and noninvasive prenatal diagnosis will be a promising development direction. WGA provides a powerful reference for the detection and diagnosis of human diseases.

## AUTHORS' CONTRIBUTIONS

XYW was responsible for the manuscript. YPL played a guiding role in reference collection and manuscript revision. HNL and WJP were mainly responsible for the revision of English grammar and expression. JR, XMZ, YMT, ZC and YD checked different sections of the manuscript. HC, NYH and SL offered revision advice. All authors read and approved the final manuscript.

## CONFLICT OF INTEREST

The authors declare no conflict of interest.

## ETHICS APPROVAL

Not applicable.

## Data Availability

Not applicable.
